# Protein methylation in mitochondria

**DOI:** 10.1016/j.jbc.2022.101791

**Published:** 2022-03-03

**Authors:** Jędrzej M. Małecki, Erna Davydova, Pål Ø. Falnes

**Affiliations:** Faculty of Mathematics and Natural Sciences, Department of Biosciences, University of Oslo, Oslo, Norway

**Keywords:** mitochondria, protein methylation, methyltransferase, bioenergetics, oxidative phosphorylation, electron transport chain, ATP synthase, 1MH, 1-methylhistidine (π-MH), 7BS, seven-β-strand, AdoHcy, *S*-adenosylhomocysteine, AdoMet, *S*-adenosylmethionine, ANT, adenine nucleotide translocase, ATPS, ATP synthase, ATPSc, ATPS c-subunit, CS, citrate synthase, eEF1A, eukaryotic elongation factor 1α, ETC, electron transport chain, ETF, electron transfer flavoprotein, KD, knock-down, KMT, lysine specific MTase, KO, knock-out, MCAD, medium chain acyl-CoA dehydrogenase, MS, mass spectrometry, MTase, methyltransferase, MTS, mitochondrial targeting sequence, OE, over-expression, OxPhos, oxidative phosphorylation, PRMT, protein arginine MTase, RF, release factor, SET, Su(var)3–9, Enhancer-of-zeste and Trithorax, TMD, transmembrane domain

## Abstract

Many proteins are modified by posttranslational methylation, introduced by a number of methyltransferases (MTases). Protein methylation plays important roles in modulating protein function and thus in optimizing and regulating cellular and physiological processes. Research has mainly focused on nuclear and cytosolic protein methylation, but it has been known for many years that also mitochondrial proteins are methylated. During the last decade, significant progress has been made on identifying the MTases responsible for mitochondrial protein methylation and addressing its functional significance. In particular, several novel human MTases have been uncovered that methylate lysine, arginine, histidine, and glutamine residues in various mitochondrial substrates. Several of these substrates are key components of the bioenergetics machinery, *e.g.*, respiratory Complex I, citrate synthase, and the ATP synthase. In the present review, we report the status of the field of mitochondrial protein methylation, with a particular emphasis on recently discovered human MTases. We also discuss evolutionary aspects and functional significance of mitochondrial protein methylation and present an outlook for this emergent research field.

## Protein methylation

**Figure 1:**
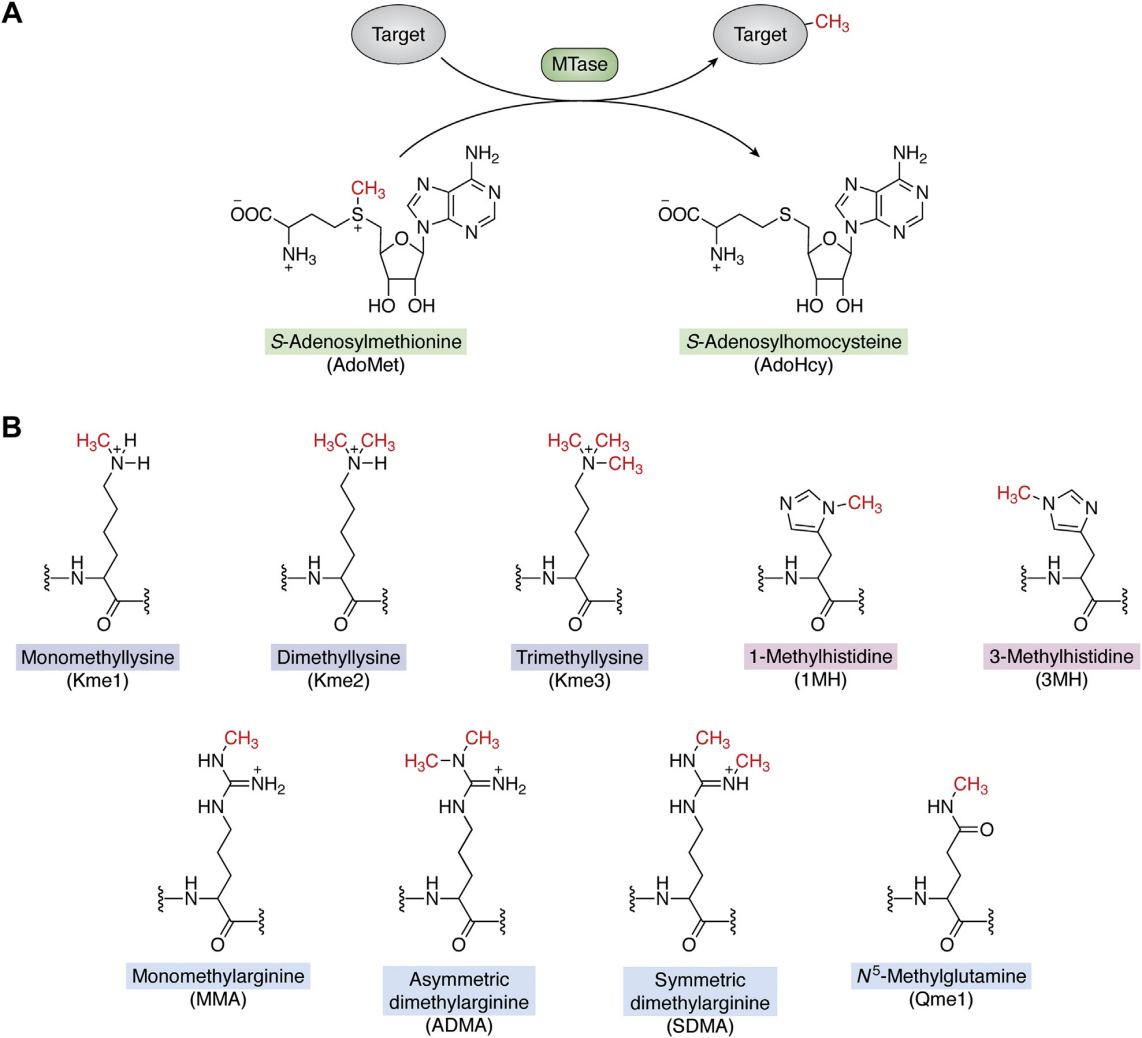
**Protein methylation by AdoMet-dependent methyltransferases (MTases).***A*, schematics of MTase-catalyzed reaction showing the methyl donor AdoMet and its demethylated counterpart AdoHcy. The transferred methyl group is shown in red. *B*, chemical structure of various methylated amino acid residues found in proteins. AdoHcy, *S*-adenosylhomocysteine; AdoMet, *S*-adenosylmethionine; MTase, methyltransferase.

Cellular proteins are commonly modified by a wide range of posttranslational modifications that serve to optimize or regulate their function. Such modifications encompass the addition of small chemical groups, *e.g.*, methylation or phosphorylation, but also complex glycosylations or the addition of entire protein domains, as in the case of ubiquitination and sumoylation. During the last two decades, it has been established that methylation is a crucial protein modification, and thousands of methylation sites have been reported in the human proteome (1). In addition, a number of methyltransferase (MTase) enzymes that mediate protein methylation have been uncovered, catalyzing the transfer of a methyl group from the universal methyl donor *S*-adenosylmethionine (AdoMet) to various substrates (Fig. 1*A*). Importantly, it has been well established that defective or altered methylation of specific proteins can cause human diseases, such as cancer and neurological disorders, thus underscoring the biological significance and medical importance of protein methylation (2, 3).

The most frequent targets of protein methylation are lysine and arginine, but also other residues, such as histidine and glutamine, can be methylated (4, 5, 6, 7) (Fig. 1*B*). Methylation increases the bulkiness of these residues and alters their hydrogen-bonding properties, but the charge remains unchanged. In the case of lysine, its ε-amino group can accept up to three methyl groups, thus yielding three distinct methylation states; mono-, di-, or trimethylation (Fig. 1*B*). Protein lysine methylation is particularly abundant in histone proteins, especially in their N-terminal, flexible tails. Histone methylations represent epigenetic marks that govern gene expression and chromatin state, and they have been studied extensively (8). The effects of histone lysine methylations are exerted through recruitment of various chromatin modifying and remodeling enzymes, which often contain so-called reader domains that in a highly specific manner recognize the methylation status of a particular lysine residue (9). There also exist several lysine demethylases that can remove such methylations; thus, histone lysine methylations are reversible and dynamic (10). Importantly, recent research has demonstrated that also a number of nonhistone proteins carry functionally important lysine methylations (7).

Arginine can be methylated on one or both nitrogen atoms in the guanidine group, thus yielding monomethylarginine, or one out of two forms of dimethylarginine: symmetric or asymmetric (Fig. 1*B*). Similar to lysine methylation, arginine methylation of histones plays a key role in epigenetic gene regulation, but a number of nonhistone proteins are also subject to arginine methylation, regulating important processes such as RNA splicing and DNA repair (4, 11). Histidine can be monomethylated at one of the two nitrogen atoms in its imidazole ring, thus producing 1-methylhistidine (1MH or π-MH) or 3-methylhistidine (3MH or τ-MH), whereas glutamine can be monomethylated at the amide group (Fig. 1*B*). The biological significance of histidine methylation has remained elusive since its discovery half a century ago, but some recent studies have provided substantial insights. The first three human histidine-specific MTases have now been discovered and functionally characterized, and it has been demonstrated that histidine methylation is abundant and pervasive (12, 13, 14, 15, 16).

## Protein methyltransferases

Bioinformatics analyses have predicted the existence of approximately 200 AdoMet-dependent MTases in humans, and many of these have been established as protein MTases (17). Based on sequence and structural homology, MTases have been divided into several classes. The so-called seven β-strand (7BS) MTases and the Su(var)3–9, Enhancer-of-zeste and Trithorax (SET) proteins form the two largest classes, together accounting for ∼90% of all human MTases (17), with the remaining MTases distributing between several minor classes, such as the SPOUT and radical-SAM MTases. The 7BS MTases contain a characteristic Rossmann-like fold, usually consisting of a seven-stranded β-sheet, and they, collectively, target a wide range of substrates, including metabolites and nucleic acids, as well as lysine, arginine, histidine, and glutamine residues in proteins (17, 18). In particular, the nine related protein arginine-(R) MTase (PRMT1-9) enzymes that are responsible for virtually all arginine methylation in human cells belong to this class, and a number of novel 7BS lysine-(K)-specific MTases (KMTs) have also been discovered in recent years (11, 19). The SET proteins contain a characteristic SET domain, which is often accompanied by a pre-SET and a post-SET region (20). In contrast to the versatile 7BS MTases, the SET proteins that have shown *bona fide* MTase activity, almost exclusively target lysines in proteins, and encompass nearly all the KMTs involved in histone methylation. Some protein MTases are highly specific, apparently targeting a single substrate protein that is recognized through structural features, exemplified by several recently discovered 7BS KMTs (21, 22, 23, 24, 25, 26, 27). Other MTases primarily recognize the local sequence surrounding the methylation site, and many of these enzymes act on a multitude of substrates, recognizing a consensus motif rather than a unique sequence. Several PRMTs and some SET-domain KMTs (*e.g.*, SMYD2 and SET7/9), as well as the recently discovered histidine methyltransferase METTL9, belong to this category (12, 28, 29).

## Mitochondrial proteins

Human mitochondria contain >1000 different proteins. The majority (∼99%) of these are nuclear-encoded and synthesized in the cytoplasm before they are imported into mitochondria, whereas only 13 proteins (∼1%) are encoded by the mitochondrial DNA and translated by the mitochondrial (55S) ribosomes in the mitochondrial matrix (30, 31). Several complex and sophisticated translocation machineries mediate mitochondrial protein import and ensure proper protein localization within the four mitochondrial compartments: the matrix, the inner and the outer mitochondrial membrane, and the intermembrane space (reviewed in (32, 33)). Moreover, mitochondrial precursor proteins contain a variety of targeting signals that mediate their recognition by the translocation apparatus and delivery to the proper destination. About 60% of mitochondrial proteins, including the majority of soluble matrix proteins and many of the inner mitochondrial membrane proteins, contain a positively charged N-terminal presequence that is usually cleaved off during import, referred to as the “classical” mitochondrial targeting sequence (MTS) (34). Several bioinformatics algorithms have been developed to predict MTSs in protein sequences, thus facilitating the identification of mitochondrial proteins. The MTS-less mitochondrial proteins contain other types of targeting signals, which are typically located internally and retained in the mature protein. In many cases, the targeting signal encompasses hydrophobic stretches that form transmembrane domains (TMDs), anchoring the protein to the inner or the outer mitochondrial membrane (32).

## Article scope

Studies on protein methylation have mainly focused on proteins found in the nucleus and the cytoplasm. Although it has been known for decades that some abundant mitochondrial proteins contain methylated residues, it was only recently that some of the corresponding MTases were identified, typically through studies aimed at unravelling the function of uncharacterized "orphan" MTases. Importantly, the identification of these MTases has also made it possible, through studies of MTase-deficient cells/organisms, to investigate the functional significance of the respective methyl modifications, and substantial novel insights have been obtained. The present review aims at presenting the current state of knowledge on mitochondrial protein methylation, with a particular focus on human MTases. The first part is devoted to the individual mitochondrial MTases, whereas the last part addresses and discusses general aspects of mitochondrial protein methylation and presents future perspectives.

## Mitochondrial protein methyltransferases and their targets

**Figure 2:**
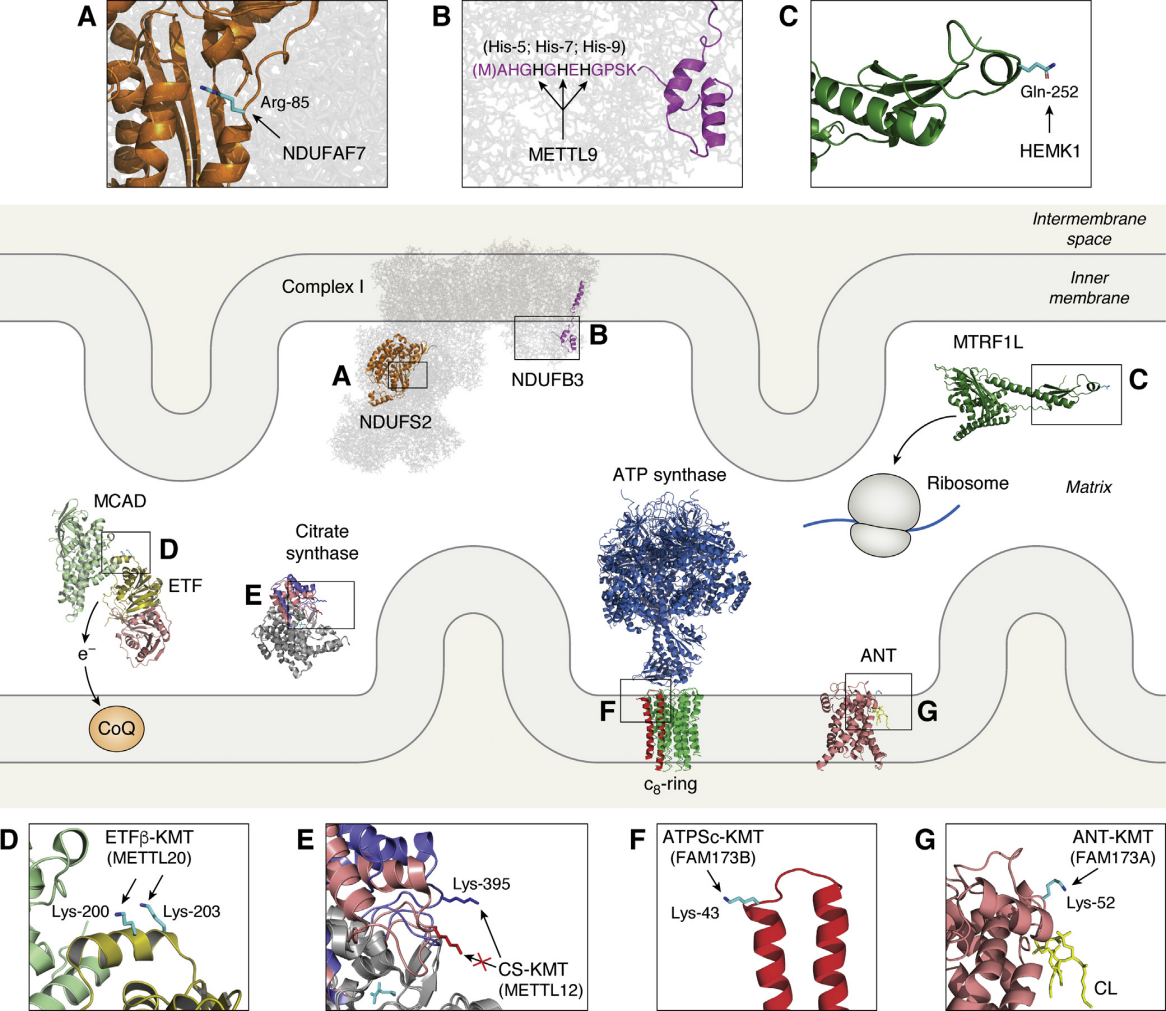
**Overview of mitochondrial protein MTases and their substrates.** Shown are the substrates of known human mitochondrial protein MTases, as well as their submitochondrial localization and the specific residues targeted. *A* and *B*, the target site(s) of NDUFAF7 on NDUFS2 (*A*) and METTL9 on NDUFB3 (*B*) are shown on the Complex I structure (generated from pdb: 5LNK). The sequence in (*B*) represents the N-terminal region of bovine NDUFB3, which was demonstrated to contain methylhistidines at positions 5, 7, and 9 (47). *C*, the methylation site in MTRF1L targeted by HEMK1 is visualized on a model generated by AlphaFold and retrieved from UniProt (ID: AF-Q9UGC7-F1). *D*, the methylation sites for ETFβ-KMT in ETFβ (*olive green*) is found in a part interacting with MCAD (*light green*) (adapted from ref. (24)). CoQ, coenzyme Q; e^-^, electron. *E*, Lys-395 in citrate synthase (CS) is methylated by CS-KMT only when the enzyme is in the “open” conformation (*blue*), but not in “closed” conformation (*red*) with bound oxaloacetate (*cyan*) (adapted from ref. (68)). *F*, the c8-ring of ATP-synthase consist of eight ATPSc monomers, one of which is shown in red and magnified to visualize the methylation site (Lys-43) (adapted from ref. (84)). *G*, in the ANT structure, a cardiolipin molecule (CL; *yellow*) is bound in the vicinity of the methylation site (Lys-52) (adapted from ref. (92)). For some of the MTases, alternative names are given in parentheses. All structural visualizations were made using PyMOL Molecular Graphics System, Version 1.3 (Schrodinger, LLC). ETF, electron transfer flavoprotein; MTase, methyltransferase

So far, seven human mitochondrial MTases have been discovered and characterized, all belonging to the 7BS MTase class, and several of these have orthologues in a wide range of organisms. In addition, Ctm1, a mitochondrial KMT only found in fungi, has been described in the budding yeast *Saccharomyces cerevisiae*. Below, individual sections are devoted to each of these eight MTases. In addition, Table 1 gives an overview of the seven mitochondrial protein MTases discovered in humans so far, whereas Figure 2 gives a graphical overview of the submitochondrial localization of their substrate proteins, indicating also the targeted residues and their orientation.

**Table 1:** Human mitochondrial protein MTases and their targets

MTase	Abbreviations (accession nr)	Substrate	Abbreviations (accession nr)	Targeted residue (modification type)	Effect of methylation	References
Protein arginine methyltransferase NDUFAF7, mitochondrial	**NDUFAF7** (NP_653337)	NADH dehydrogenase [ubiquinone] iron-sulfur protein 2, mitochondrial	**NDUFS2** (NP_001364227)	**Arg-85**^a^ (SDMA)	Promotes Complex I assembly. NDUFAF7 variants associated with pathologic myopia.	(38, 39, 40, 44)
Methyltransferase-like protein 9	**METTL9** (NP_057109.3)	NADH dehydrogenase [ubiquinone] 1 beta subcomplex subunit 3	**NDUFB3** (NP_001244031.1)	**His-5, His-7, His-9**^b^ (1MH)	Promotes Complex I-dependent mitochondrial respiration.	(12, 47)
MTRF1L release factor glutamine methyltransferase	**HEMK1** (NP_001304780)	Peptide chain release factor 1-like, mitochondrial	**MTRF1L** (NP_061914)	**Gln-252**^b^ (Qme1)	Promotes proper termination of translation.	(53)
Electron transfer flavoprotein beta subunit lysine N-methyltransferase	**ETFβ-KMT** **METTL20** (NP_001129335.1)	Electron transfer flavoprotein subunit beta	**ETFβ** (NP_001976.1)	**Lys-200, Lys-203**^b^ (Kme1, Kme2, Kme3)	Decreases ETF-dependent electron transfer.	(24, 61, 63)
Citrate synthase lysine N-methyltransferase, mitochondrial	**CS-KMT** **METTL12** (NP_001036694.1)	Citrate synthase, mitochondrial	**CS** (NP_004068.2)	**Lys-395**^b^ (Kme1, Kme2, Kme3)	Decreases CS activity.	(68, 69)
ATP synthase subunit c lysine N-methyltransferase	**ATPSc-KMT** **FAM173B** (NP_954584.2)	ATP synthase F(0) complex subunit c, mitochondrial	**ATPSc** (NP_001002027.1, NP_001002031.1 and NP_001002258.1 precursors produce identical mature protein)	**Lys-43**^a^ (Kme3)	Promotes ATP synthase assembly and mitochondrial respiration. ATPSc-KMT variants associated with chronic pain.	(83, 84, 85)
Adenine nucleotide translocase lysine N-methyltransferase	**ANT-KMT** **FAM173A** (NP_076422.1)	Adenine nucleotide translocase 1, -2, -3	**ANT1** (NP_001142.2), **ANT2** (NP_001143.2), **ANT3** (NP_001627.2)	**Lys-52**^b^ (Kme3)	Decreases mitochondrial respiration.	(92)

Presented data refer to *Homo sapiens* proteins. Accession numbers according to NCBI database are given for main protein isoforms (1 or a).

a amino acid numbering based on sequence of mature protein. See Figure 1 for abbreviations of modification types.

b amino acid numbering based on sequence of precursor protein.

Of note, in databases on protein methylation such as PhosphoSitePlus, a nonprocessed protein, *i.e.*, the relevant UniProt/SwissProt entry, is typically used as the basis for amino acid numbering. In contrast, older studies of mitochondrial protein methylation have typically used numbering corresponding to the mature, processed protein. Here, we have used "precursor numbering" for some methylation targets and "mature numbering" for others (and in some cases also indicated both types of numbering), based on its use in the scientific literature.

### NDUFAF7-mediated arginine methylation of subunit NDUFS2 from Complex I

Mammalian Complex I, also denoted as NADH:ubiquinone oxidoreductase, is part of the mitochondrial electron transport chain (ETC) and catalyzes oxidation of NADH in the mitochondrial matrix with concomitant reduction of the mitochondrial ubiquinone (coenzyme Q) pool. This process is coupled to the extrusion of four protons from the mitochondrial matrix into the intermembrane space and contributes to the generation of the proton motive force used by the mitochondrial ATP synthase (ATPS) to produce ATP during oxidative phosphorylation (OxPhos). Complex I forms an L-shaped structure, with one hydrophobic arm embedded in the inner mitochondrial membrane and a second hydrophilic arm protruding into the mitochondrial matrix (Fig. 2, *A* and *B*) (35). The catalytic core of Complex I is composed of several mitochondrial- and nuclear-encoded proteins, including the NDUFS2 subunit, which is localized at the junction between the two arms (Fig. 2*A*). In addition, Complex I contains several so-called “supernumerary” or “accessory” subunits, such as NDUFAF7, which are not directly involved in the catalytic mechanism, but are required for proper biogenesis and assembly of the complex (reviewed in (36)).

NDUFAF7/MidA was originally identified in the slime mold *Dictyostelium discoideum* as an evolutionary conserved protein of α-proteobacterial origin, whose deletion resulted in reduced ATP synthesis and Complex I activity (37, 38). Bioinformatics analysis predicted NDUFAF7 to contain a classical MTS, and its mitochondrial localization was confirmed in several independent studies (38, 39, 40). Genetic screening and direct pull-down assays demonstrated that human and *Dictyostelium* NDUFAF7 interact directly with NDUFS2, and bioinformatics analysis and structural modeling indicated NDUFAF7 to contain a 7BS MTase domain (17, 38). Interestingly, NDUFS2 contains an evolutionary conserved arginine (Arg-85 in humans; mature numbering), which was found to be symmetrically dimethylated in several eukaryotic organisms (41). Rhein *et al*., (39) demonstrated that Arg-85 in NDUFS2 from human osteosarcoma cells was fully dimethylated, but its methylation was strongly reduced after prolonged NDUFAF7 knock-down (KD), indicating that NDUFAF7 is the arginine-specific MTase responsible for methylation of NDUFS2 at Arg-85 *in vivo*. Additionally, evidence was provided for *in vitro* arginine methylation of NDUFS2-derived peptide by recombinant NDUFAF7 from *Dictyostelium* (42). Of note, NDUFAF7 represents an arginine-specific MTase unrelated to other PRMTs.

NDUFS2 is located near the catalytic center of Complex I, in close proximity to some of the iron-sulfur clusters, where it contributes to ubiquinone binding (35). Accordingly, knock-out (KO) or KD of the MTase NDUFAF7 in human cells affected Complex I assembly and respiration (39, 40). In *Dictyostelium*, NDUFAF7 KO affected Complex I-dependent respiration and caused phototaxy and growth defects, which were rescued by wild-type NDUFAF7, but not by an MTase-deficient mutant (38, 42). NDUFAF7 KD in zebrafish caused impaired Complex I assembly, delayed hatching and morphological abnormalities, whereas NDUFAF7 KO was embryonically lethal in mice (40). Interestingly, recent studies indicate that NDUFAF7 variants may be associated with pathologic myopia in humans (43, 44). Overall, these studies highlight the importance of NDUFAF7-mediated NDUFS2 methylation in various physiological processes in several different organisms.

### METTL9-mediated histidine methylation of subunit NDUFB3 from Complex I

NDUFB3 is one of the accessory subunits of the mitochondrial ETC Complex I. It consists of a C-terminal domain, which forms an alpha helix spanning the inner mitochondrial membrane, and a hydrophilic N-terminal region facing the matrix (Fig. 2*B*). The N-terminal region of NDUFB3 is crucial for Complex I assembly and function, and a homozygous mutation (Trp22Arg) in this region was found to cause mitochondrial disease in humans. Affected patients showed short stature and distinctive facial appearances and, to varying degrees, defects in metabolism and Complex I assembly (45, 46). The N-terminus of vertebrate NDUFB3 contains a stretch of alternating histidines (HxHxH….), varying in length between species and containing from two to nine alternating histidines, with five histidines, *i.e.*, HxHxHxHxH being present in humans (12). MS analysis of the corresponding fragment in bovine NDUFB3, which contains four alternating histidines, revealed that several of these histidines were monomethylated in various combinations, with the second and the third histidine of the motif being methylated most frequently, and the first histidine remaining unmodified in all cases (47).

The hitherto uncharacterized 7BS MTase METTL9 was recently found to methylate histidines at HxH motifs in several mammalian proteins, including NDUFB3 (12). Amino acid analyses of protein hydrolysates revealed that METTL9 exclusively introduces 1MH both *in vitro* and *in vivo*. Importantly, histidine methylation of human NDUFB3 was completely abolished in METTL9 KO cells, thus firmly demonstrating that METTL9 is the MTase responsible for NDUFB3 methylation (12). However, it remains unclear whether NDUFB3 methylation takes place before or after its mitochondrial import, as METTL9 is found in multiple cellular compartments, including the cytosol and mitochondria (12). Interestingly, METTL9 KO cells showed diminished Complex I-dependent mitochondrial respiration, which was restored to WT levels after complementation with enzymatically active METTL9, indicating that histidine methylation of NDUFB3 is important for Complex I function (12).

METTL9 has a broad organismal distribution, with homologues found in all metazoans, but also in some protists and unicellular algae. However, it is likely that METTL9-mediated methylation of NDUFB3 is restricted to vertebrates only, which are the only organisms with HxH motifs at the N-terminus of NDUFB3.

### HEMK1/Mtq1p-mediated glutamine methylation of translation release factors

Translation release factors (RFs) are conserved proteins universally present in all kingdoms of life, acting as tRNA-mimics that recognize stop codons and terminate mRNA translation. Mitochondrial RFs show high sequence homology to their bacterial counterparts, reflecting the bacterial evolutionary origin of mitochondria. All RFs contain a conserved GGQ motif that interacts with the ribosomal peptidyl transferase center (48) and is subject to glutamine monomethylation in many organisms. In bacteria, the 7BS MTase HemK (also referred as PrmC) targets the release factors RF1 and RF2 (49, 50). Correspondingly, sequence homologues of HemK catalyze methylation of eukaryotic mitochondrial release factors. In yeast, the HemK-homologue, Mtq1p, was found to localize to mitochondria and to mediate both *in vitro* and *in vivo* monomethylation of the mitochondrial release factor Mrf1p at Gln-287 (precursor numbering), located within the GGQ motif (51, 52). Similarly, the human MTase HEMK1 was shown to localize to mitochondria and to monomethylate Gln-252 of the canonical mitochondrial release factor MTRF1L (Fig. 2*C*) (53, 54). Another human mitochondrial RF, MTRF1, which recognizes Arg codons (AGG and AGA) that have been reassigned to stop codons, is highly homologous to MTRF1L (55). Thus, it is likely that also MTRF1 is subject to HEMK1-mediated methylation, but this remains to be demonstrated experimentally.

Since the GGQ motif is important for interaction with the ribosomal peptidyl transferase center, one may anticipate that ablation of its glutamine methylation may affect mitochondrial translation (48). Also, since nearly all of the 13 proteins encoded by mitochondrial DNA are subunits of mitochondrial ETC complexes, effects on mitochondrial translation will likely have an impact on mitochondrial respiration as well. In agreement with this, *Δmtq1* yeast showed increased stop codon read-through and grew slower than the wild-type strain in nonfermentable media containing ethanol or glycerol as the only carbon source (51). Similarly, siRNA-mediated depletion of HEMK1 in human HeLa cells resulted in decreased mitochondrial translation (53).

In summary, HemK orthologues appear to mediate glutamine methylation of mitochondrial RFs across the eukaryotic kingdom, and such methylation seems to ensure accurate and efficient mRNA translation in mitochondria. However, further investigations, such as structural studies on translating ribosomes, are required to reveal the corresponding molecular mechanisms.

### ETFβ-KMT (METTL20)-mediated lysine methylation of the β-subunit of electron transfer flavoprotein

Electron transfer flavoprotein (ETF) is an α/β heterodimer that localizes to the mitochondrial matrix and acts as a mobile carrier of electrons from several FAD-containing dehydrogenases to ETF:quinone oxidoreductase, which mediates reduction of the mitochondrial ubiquinone pool (reviewed in (56)). These ETF-dependent dehydrogenases mediate *e.g.* β-oxidation of fatty acids and degradation of choline and various amino acids (reviewed in (57)), thus making ETF a major provider of electrons for the ubiquinone pool of the ETC (58).

Based on sequence similarity, METTL20 was identified as one of ten human members of the so-called MTase Family 16, which primarily encompasses 7BS KMTs that target nonhistone proteins (21, 22, 25, 27, 59, 60). Bioinformatics analysis suggested METTL20 to contain a cleavable MTS located at its N-terminus and accordingly, METTL20 was shown to localize to mitochondria (24, 39, 61). Two independent studies demonstrated that human METTL20 specifically targets the β-subunit of ETF (ETFβ), thus representing the first discovered human mitochondrial KMT (24, 61). The first study identified ETFβ as a binding partner of METTL20 (61), whereas the second study used an activity-based purification to identify ETFβ as the substrate of recombinant METTL20 (24), and both studies revealed that METTL20 methylates ETFβ on two adjacent residues, Lys-200 and Lys-203 (precursor numbering). The extent of ETFβ methylation varied between cell types, with Lys-200 and Lys-203 typically being di- or trimethylated, thus producing a complex mixture of methylation states, with each ETFβ molecule carrying up to six methyl groups. Importantly, siRNA-mediated knockdown of METTL20 led to diminished ETFβ methylation, whereas methylation was increased by METTL20 overexpression (OE) (24, 61), thus firmly establishing METTL20 as the MTase responsible for ETFβ methylation *in vivo*. Based on these findings, METTL20 was renamed ETFβ-KMT (gene name *ETFBKMT*) (24).

Both methylated lysines in ETFβ are located in close proximity to the so-called “recognition loop” that interacts with medium chain acyl-CoA dehydrogenase (MCAD) (Fig. 2*D*) (62) and likely mediates interaction with other ETF-dependent dehydrogenases. Indeed, methylated ETFβ showed, relative to its unmethylated counterpart, decreased ability to extract electrons from various ETFβ-dependent dehydrogenases *in vitro* (24), and accordingly, mitochondrial extracts from ETFβ-KMT KO mice displayed higher ETF activity than WT extracts (63). In line with this, the KO mice showed increased heat production and O_2_ consumption when fed a ketogenic diet and also displayed increased tolerance to low temperature under fasting (63). Taken together, these findings indicate that ETFβ-KMT–mediated methylation decreases ETF activity and may play an important role in regulating metabolic processes, such as the β-oxidation of fatty acids.

ETFβ-KMT shows a scattered organismal distribution, with putative orthologues found in all chordates, but only in selected invertebrates, such as the nematode *Caenorhabditis elegans* (24). Strikingly, METTL20 homologues are also found in a few bacteria, especially in α-proteobacteria, which are considered the evolutionary precursors of mitochondria (64). Also, such bacterial homologues were shown to methylate ETFβ (64), and ETFβ-KMT/METTL20 therefore represents the first KMT found to catalyze the same reaction in bacteria and humans.

### CS-KMT (METTL12)-mediated lysine methylation of citrate synthase

Citrate synthase (CS) resides in the mitochondrial matrix and catalyzes the rate-limiting step of the Krebs cycle, namely the synthesis of citrate from acetyl-CoA and oxaloacetate. It has been known for four decades that Lys-395 (precursor numbering) in mammalian CS is modified to trimethyllysine (65), but the responsible MTase remained elusive until very recently.

Based on sequence similarity, METTL12 was identified as one of four human members of a small family of 7BS MTases, which otherwise encompasses KMTs targeting eukaryotic elongation factor 1α (eEF1A) (23, 66, 67). Bioinformatics analysis suggested METTL12 to contain a cleavable, N-terminal MTS (39, 68), and accordingly, METTL12 was shown to localize to mitochondria (68). Recently, two parallel studies identified METTL12 as the long-sought MTase responsible for methylation of CS at Lys-395 (68, 69). The first study showed that METTL12 KO resulted in loss of CS methylation in cells (69), while the second study identified CS as the substrate of recombinant METTL12, and proved that complementation of METTL12 KO cells with an enzyme-active METTL12, but not with an enzyme-dead mutant, resulted in restoration of Lys-395 trimethylation (68). Together, the two studies firmly established METTL12 as the enzyme responsible for CS methylation *in vivo*, and consequently, METTL12 was renamed CS-KMT (gene name *CSKMT*) (68).

Regulation of CS has been extensively investigated, and it is generally accepted that the overall rate of CS-catalyzed reaction is dictated by the availability of its two substrates, oxaloacetate and acetyl-CoA, which are typically present in mitochondria at limiting concentrations and below CS saturation levels (reviewed in (70, 71)). CS is also inhibited by its product, citrate, and a downstream product of the Krebs cycle, succinyl-CoA (72, 73). Recent work suggests that CS activity may also be influenced by CS-KMT-mediated methylation at Lys-395 (68), which is localized in the vicinity of the CS active site (Fig. 2*E*) (74). It was shown that incubation of CS with wild-type CS-KMT, in the presence of AdoMet, decreased CS activity, but no such effect was observed when AdoMet was omitted or an enzyme-dead mutant of CS-KMT was used (68). Binding of oxaloacetate induces CS to adopt a “closed state” conformation, where the segment encompassing Lys-395 is reoriented (Fig. 2*E*) (75), and methylation of CS by CS-KMT was shown to be strongly inhibited by oxaloacetate (68). Moreover, MTases are generally inhibited by *S*-adenosylhomocysteine (AdoHcy), the by-product of MTase reactions (Fig. 1*A*), and CS-KMT was found to be particularly sensitive to such inhibition (68). Thus, the observed inhibition of CS methylation by oxaloacetate and AdoHcy *in vitro* may suggest that methylation at Lys-395 is modulated in response to the metabolic state also *in vivo*. In line with this notion, CS methylation status was found to vary depending on the cell type; Lys-395 showed full trimethylation in various pig organs and several human cell lines, whereas methylation levels were lower in other cell lines (68, 69).

CS-KMT orthologues show a somewhat scattered evolutionary distribution and are present mainly in vertebrates, but also in some invertebrates (68). Among mammals, CS-KMT is found in human, cow, pig, and a subset of rodents, such as guinea pig, but is absent in rat and mouse. This suggests that CS methylation is not strictly required for its function, but rather serves an optimizing or regulatory role that is beneficial under certain conditions.

### ATPSc-KMT (FAM173B)-mediated lysine methylation of the ATP synthase c-subunit

Mitochondrial Complex V, primarily known as ATP synthase (ATPS), mediates the synthesis of ATP from ADP and phosphate during OxPhos, driven by the proton-motive force generated by transport of electrons through the ETC (76). ATPS consists of two regions, F_1_ and F_O_, where the catalytic part, F_1_, faces the mitochondrial matrix, and F_O_ traverses the inner mitochondrial membrane. In metazoans, the central core of F_O_ is composed of eight membrane-embedded ATPS c-subunits (ATPSc) assembled into the so-called "c8-ring" (Fig. 2*F*), which, together with proteins of the so-called “central stalk,” constitute the rotary element of ATPS (77). In humans, three distinct genes (*ATP5MC1*, *ATP5MC2*, and *ATP5MC3*) encode slightly different ATPSc precursors that give rise to an identical mature protein. Several independent reports showed that ATPSc is invariably trimethylated at Lys-43 (mature numbering) in metazoans (78, 79, 80).

A broad-specificity 7BS KMT, denoted as "aKMT," is found in certain archaeabacteria, such as *Sulfolobus islandicus*, and it was noted that a distant aKMT sequence homologue, denoted as FAM173B, is present in mammals (19, 81, 82). Interestingly, mammalian FAM173B was found to be a mitochondrial protein, localized specifically to the cristae (83). FAM173B consists of a stretch of N-terminal nonconserved sequence, followed by a putative TMD, followed by a conserved "pre-MTase" domain, and the MTase domain. Notably, the N-terminal sequence and the TMD were dispensable for mitochondrial targeting of FAM173B, and the pre-MTase domain was by itself able to target green fluorescent protein to mitochondria (84). FAM173B appears not to be processed during mitochondrial import, indicating that the entire N-terminal region represents an atypical noncleavable MTS, with the TMD likely functioning as a membrane anchor. Importantly, it was found by Western blotting with methyllysine-specific antibodies that FAM173B KO cells were deficient in methylation of a single protein of ∼8 kDa in size, identified as ATPSc (84). Trimethylation of Lys-43 in ATPSc was completely abolished in KO cells, but was fully restored after complementation with enzymatically active FAM173B (84). This firmly establishes FAM173B as the long-sought MTase responsible for methylation of ATPSc *in vivo*, and accordingly, FAM173B was renamed ATPSc-KMT (gene name *ATPSCKMT*) (84).

ATPSc-KMT-deficient cells showed accumulation of intermediates of the ATPS complex, indicating defects in Complex V assembly (84). Moreover, these cells also displayed decreased ATP synthesis and O_2_ consumption related to OxPhos (84). Interestingly, a genome-wide association study implicated ATPSc-KMT in development of chronic pain in humans (85), and a subsequent study showed that ATPSc-KMT KD abrogated chronic pain in a mouse model, whereas ATPSc-KMT OE promoted pain (83). ATPSc-KMT OE in neurons increased mitochondrial hyperpolarization and production of reactive oxygen species (83). It remains unclear, however, how changes induced by ATPSc-KMT OE are linked to Lys-43 methylation, since ATPSc is generally found to be fully trimethylated under non-OE conditions, *i.e.*, in various tissues/cells from several different species (84).

Lys-43 is evolutionary conserved and located on a solvent-exposed loop facing the matrix side of the inner mitochondrial membrane (Fig. 2*F*). Lys-43 in ATPSc has been shown to be involved in the binding of the anionic lipid cardiolipin, which is abundant in the inner mitochondrial membrane, and considered an essential component of the ATPS complex (86). Thus, one may speculate that Lys-43 methylation somehow optimizes the ATPSc/cardiolipin interaction, thus ensuring efficient assembly and function of the ATPS complex.

Sequence homologues of ATPSc-KMT are found throughout the animal kingdom, in agreement with the apparent ubiquitous presence of trimethyllysine in ATPSc from metazoans (80). The ATPSc-KMT orthologue from the nematode *C. elegans* was shown to methylate human ATPSc at Lys-43 when introduced into ATPSc-KMT KO cells, indicating that also nonvertebrate homologues of ATPSc-KMT target ATPSc (84).

### ANT-KMT (FAM173A)-mediated lysine methylation of adenine nucleotide translocase

The mitochondrial adenine nucleotide translocase (ANT), alternatively referred to as the ADP/ATP translocase or the ADP/ATP carrier, is a transmembrane protein residing inside the inner mitochondrial membrane, where it forms a protein channel (87, 88). ANT promotes continuous synthesis of ATP by the mitochondrial ATPS through constant exchange of ATP synthesized in the mitochondrial matrix for ADP present in intermembrane space. Four paralogous genes (ANT1, -2, -3, and -4) encode four different ANT isoforms in mammals (89), and three of these, namely ANT1, -2, and -3, have been reported to be trimethylated at Lys-52 (precursor numbering), a conserved residue present in all ANT isoforms and located on a solvent-exposed loop facing the matrix side of the inner mitochondrial membrane (Fig. 2*G*) (90, 91).

In addition to ATPSc-KMT (FAM173B) described in the preceding section, which is ubiquitously present in all *Metazoa*, vertebrates have an additional paralogue, FAM173A (84). The two proteins show sequence homology throughout their respective sequences, and it was shown also for FAM173A that its N-terminal region contains an atypical, noncleavable MTS responsible for mitochondrial localization, and with membrane anchoring putatively mediated by the hydrophobic TMD (84, 92). Similarly as for ATPSc-KMT, using methyllysine-specific antibodies, it was shown that a ∼32 kDa protein species was devoid of methylation in FAM173A KO cells. This protein species was identified as a mixture of ANT2 and ANT3 (92), which both showed trimethylation at Lys-52 in cells expressing enzymatically active FAM173A, but not in FAM173A-deficient cells. Moreover, it was demonstrated that ANT1 and ANT2 are fully trimethylated at Lys-52 in a variety of rat tissues. Collectively, these data firmly establish FAM173A as the MTase responsible for methylation of ANT *in vivo*, and consequently, FAM173A was renamed ANT-KMT (gene name *ANTKMT*) (92).

Since ADP/ATP exchange across the inner mitochondrial membrane is required for ATP synthesis, ANT methylation may conceivably affect mitochondrial respiration, which is coupled to ATP synthesis. Indeed, mitochondrial respiration linked to ATP synthesis by OxPhos was increased in ANT-KMT KO cells and restored to WT levels after complementation with enzyme-active FAM173A, but not with the enzyme-dead mutant (92). Interestingly, ANT tightly binds cardiolipin (93), and one of the three cardiolipin molecules found in ANT crystal structures is located in the vicinity of Lys-52. Moreover, it was reported that cardiolipin binding to ANT influenced its self-association and interaction with other proteins (94, 95, 96). Therefore, one may speculate that ANT-KMT-mediated methylation of ANT at Lys-52 evolved to optimize its interaction with cardiolipin and modulate its cardiolipin-dependent activities.

As outlined above, vertebrates possess two similar paralogues, ATPSc-KMT and ANT-KMT, both targeting ring-forming proteins/structures in the inner mitochondrial membrane, with the methylated lysine facing the matrix side and being involved in cardiolipin binding (Fig. 2, *F* and *G*). In contrast, nonvertebrate animals only have one such protein, targeting ATPSc (84, 92), and it is therefore reasonable to assume that ATPSc-KMT represents the primordial enzyme, from which the vertebrate-specific ANT-KMT evolved after a gene duplication event (92).

### Ctm1p-mediated lysine methylation of cytochrome c in fungi

Cytochrome c is a highly conserved protein universally present in all eukaryotes, containing a covalently attached heme group, and its function depends on its intracellular localization. Typically, cytochrome c localizes to the mitochondrial intermembrane space and acts as mobile carrier for the ETC, transferring electrons between Complex III (also known as the cytochrome bc_1_ complex or ubiquinol:cytochrome c oxidoreductase) and Complex IV (also known as cytochrome c oxidase). However, in higher animals, various cellular stresses can induce the release of cytochrome c from mitochondria into cytosol, thus triggering the process of programmed cell death, also known as apoptosis.

Many plants and fungi contain cytochrome c with methylated lysine(s) (97). In yeast, cytochrome c (Cyc1p) is trimethylated at Lys-78 (precursor numbering), whereas the corresponding residue in mammals, referred to as Lys-72 (mature numbering), is unmethylated (98). The responsible enzymatic activity, cytochrome c-KMT, was partially purified from various sources and shown to mediate methylation of cytochrome c of fungal and mammalian origin (99, 100), but it took nearly two decades until the corresponding gene, *CTM1*, was identified in yeast (101, 102). It was demonstrated that cytochrome c from the *ΔCtm1* strain was completely unmethylated, and recombinant Ctm1p was shown to specifically target unmethylated cytochrome c (from mammals or *ΔCtm1* yeast) *in vitro* (102). Moreover, Lys-78 in Cyc1p was confirmed as the Ctm1p target site by mutational analysis (102) and mass spectrometry (MS) (103). Collectively, these studies firmly established Ctm1p as the KMT responsible for methylation of yeast cytochrome c *in vivo* (102, 103).

Unlike the other MTases described in this review, which are all 7BS MTases, Ctm1p belongs to the SET-domain class of MTases, which primarily encompasses KMTs involved in histone methylation. Ctm1p is localized to the cytoplasm, indicating that methylation of cytochrome c occurs in the cytoplasm, prior to mitochondrial import (102, 104). Apocytochrome c, *i.e.*, cytochrome c without the heme, is the preferred substrate of Ctm1p, and methylation of cytochrome c was found to enhance both its mitochondrial import and its interaction with the cytochrome c heme lyase, Cyc3p, which is responsible for heme attachment (103, 105). Since heme attachment promotes the mitochondrial import of cytochrome c, these results are compatible with a model where methylation promotes cytochrome c heme attachment and mitochondrial import through enhancing the interaction between cytochrome c and Cyc3p. Several studies reported that methylation affected various biochemical properties of cytochrome c (106, 107, 108, 109), but the biological significance of these results remains unclear, since *Δctm1* yeast strain has no observable functional defects (102). Also, the absence of cytochrome c methylation in *Metazoa* suggests that it is not essential for cytochrome c function in mitochondrial respiration.

## General discussion

### Functional roles of mitochondrial protein methylation

Typically, methylated residues in mitochondrial proteins are located at the protein surface (Fig. 2) and often interact with other molecules, suggesting that methylation may optimize or modulate such interactions. For example, the methylated residue Gln-252 in MTRF1L is part of a conserved GGQ-motif that interacts with the ribosomal peptidyl transferase center (48), whereas the methylated residues Lys-200 and Lys-203 in ETFβ are part of the “recognition loop” that interacts with MCAD and, likely, with other ETF-dependent dehydrogenases (62). Also, it was suggested that methylation of Arg-85 in NDUFS2 is important for binding of another subunit, NDUFS7, during early steps of Complex I assembly (39, 42), and that methylation of Lys-43 in ATPSc may modulate cardiolipin binding (110).

In the context of nuclear proteins, such as histones and transcription factors, methylations often play regulatory roles, and may therefore be portrayed as tunable "switches" governing transcriptional activity and/or chromatin state in a dynamic manner. These methylation "marks" are specifically recognized by reader domains that are part of multidomain proteins that modify chromatin through various enzymatic activities, and, in the case of lysines, methylation is dynamic, as it can be reversed by the action of various demethylases. In contrast, many of the cytosolic lysine methylations that have been discovered in recent years appear static and constant, and these may rather be likened with "nuts and bolts," playing important roles in optimizing protein function, but without being dynamic or exerting a regulatory function. Several of the mitochondrial methylations apparently belong to this latter category, for example, lysine methylation of ANT and ATPSc, which were both found in a fully trimethylated state in all tissues and cells examined (84, 92).

To our knowledge, there are no indications that protein demethylases exist in mitochondria. Almost all histone demethylases belong to the so-called JmjC-domain protein family, and none of these enzymes have been observed in mitochondria. The AlkB homologues (ALKBH), which are similar to the bacterial DNA repair protein AlkB, represent another group of vertebrate demethylases, belonging to the same superfamily as the JmjC-domain proteins (111). Actually, two of these, ALKBH1 and ALKBH7 localize to mitochondria, but they modify tRNA bases (112, 113, 114). However, it should be mentioned that a single study reported that ALKBH1 targets proteins, but this has not been confirmed by others (115).

Interestingly, lysine methylation of CS and ETFβ, as well as METTL9-mediated histidine methylation of NDUFB3, were shown to be substoichiometric in mammalian cells, with a substantial and variable proportion of lower methylation states (24, 68). This may be due to the reversal of methylation by yet uncovered demethylases, but could also simply reflect a delay in the methylation of newly synthesized proteins, due to limited MTase capacity. In a third scenario, these methylations may be truly dynamic and regulated, but solely at the level of the MTase, with demethylation occurring by the passive mechanism of protein turnover.

In recent years, substantial cross talk between protein modification and cellular metabolism has been demonstrated. For example, levels of protein acetylation are strongly influenced by the levels of the acetyl donor, acetyl-CoA, which is generated by key metabolic processes such as fatty acid breakdown and the citric acid cycle (116). Similarly, certain cellular methylations are particularly sensitive to alterations in the levels of the methyl donor AdoMet and its unmethylated counterpart AdoHcy, which may act as an inhibitor of enzymatic methylation reactions (117). Both AdoMet and AdoHcy take part in the so-called one-carbon (1C) cycle, which is intertwined with several metabolic pathways (118). Interestingly, methylation of CS by CS-KMT appeared to be particularly sensitive to inhibition by AdoHcy, potentially representing a regulatory mechanism whereby changes in the AdoMet/AdoHcy ratio may modulate CS activity, which was found to be diminished by methylation (68). Also ETFβ methylation shows potential links to one-carbon metabolism, since ETF-dependent dehydrogenases are involved in the metabolism of the 1C metabolites sarcosine and dimethylglycine (56).

In several cases, methylation was found to affect the activity of a metabolic protein, thus influencing metabolite levels; for example, methylation diminished CS and ETFβ activity (24, 68). In the case of CS, the converse also occurred, namely that the metabolite influenced methylation, as CS adopts a "closed" conformation in the presence of its substrate oxaloacetate, thereby blocking CS-KMT-mediated methylation (68). Thus, a self-regulatory circuit is potentially formed, where oxaloacetate build-up inhibits CS methylation, leading to increased levels of unmethylated (more active) CS and accelerated oxaloacetate consumption. However, these features of CS and CS-KMT were only observed *in vitro*, and it remains to be established whether this circuit is operative and significant *in vivo*. As discussed in this section, some of the mitochondrial protein methylation events display low occupancy and interesting variations, and there appears to be cross talk between mitochondrial methylation and cellular metabolism. However, more research is required to establish the extent to which mitochondrial protein methylation actively regulates metabolism *in vivo*.

### The mitochondrial methylproteome

During the last decade, several high-throughput MS studies have revealed thousands of methylation sites in mammalian proteomes, focusing mainly on lysines and arginines (29, 91, 119, 120, 121, 122, 123, 124). Data from such studies have been compiled in the PhosphoSitePlus database (1), which, for humans, currently encompasses ∼16,000 methylation sites distributed between ∼5500 proteins. To investigate the extent of mitochondrial protein methylation, we extracted from PhosphoSitePlus the methylated human proteins that are annotated as mitochondrial in the UniProt database (note that some of these proteins have additional subcellular localizations). This retrieved 385 methylated mitochondrial human proteins, containing 775 methylation sites (Table 2). Comparison of the mitochondrial subproteome to the total proteome revealed a similar fraction of proteins that are methylated (27–31%), and a comparable number of methylations per (methylated) protein (between two and three), as well as a similar ratio of Arg to Lys methylations (∼2:1) (Table 2). This analysis suggests that mitochondrial protein methylation might be extensive and comparable to the cellular average. However, it is important to point out that data from high-throughput studies should be approached with caution, as the false discovery rates for protein methylation are typically very high (125). In particular, many of the putative monomethylation events may result from incorrect assignment of methylpeptide MS spectra or artefactual methyl-ester formation occurring during sample preparation (125).

**Table 2:** Protein methylations in human mitochondria (from PhosphoSitePlus)

Category	Total	Mitochondrial
**All proteins**	20,386	1234
**Methylated proteins**	5447 (26.7%^a^)	385 (31.2%^a^)
**Methylations**	15,919	775
**Methylations per protein**	2.9	2.0
**Arg methylations**	10,806 (67.9%^b^)	525 (67.7%^b^)
- Rme1	0.86	0.94
- Rme2	0.14	0.06
**Lys methylations**	5101 (32.0%^b^)	248 (32.0%^b^)
- Kme1	0.83	0.80
- Kme2	0.11	0.11
- Kme3	0.06	0.09
**Other methylations**	12 (0.1%^b^)	2 (0.3%^b^)

Data on human protein methylation were downloaded from PhosphoSitePlus (in Sept. 2021). Methylations were designated as "mitochondrial" when the corresponding protein is annotated with a mitochondrial localization in UniProt. For Arg and Lys methylations, the fraction constituted by each individual methylation state is indicated.

a of all proteins.

b of all methylations.

Besides the high-throughput studies described above, methylation of a few mammalian mitochondrial proteins has been discovered through low-throughput studies focusing on a single abundant protein or protein complex. Somewhat strikingly, almost all the human MTases described in previous sections were found to mediate methylation events originally identified in low-throughput studies, *i.e.*, those on NDUFS2, CS, ATPSc, ANT, and NDUFB3 (41, 47, 65, 79, 90). Common for these low-throughput studies is that they typically identified sites that contained more than one methyl group (*i.e.*, dimethyl-Arg or trimethyl-Lys), and that methylation was of high occupancy, *i.e.*, that the methylated, but not the unmethylated form was detected. In contrast, the high-throughput studies typically used affinity-based enrichment of methylated sequences (using methylation-specific antibodies), which would also identify low occupancy methylations. Moreover, more than 80% of the methylation events discovered in these high-throughput studies were monomethylations (Table 2). One may therefore speculate that many of these methylation events are of low occupancy and without biological significance, either representing artefactual methylations (see above) or resulting from spurious activity of broad-specificity protein MTases. No mitochondrial broad-specificity protein MTases have been described, but it is clearly possible that cytoplasmic MTases methylate mitochondrial proteins prior to their import into mitochondria. Candidate MTases may be the SET-domain KMTs SMYD2 and SET7/9, as well as several of the PRMT enzymes. Also, one should not exclude the possibility that some low occupancy methylations may result from nonenzymatic methyl transfer from the methyl donor AdoMet.

Despite the high number of human mitochondrial protein methylations that can be extracted from PhosphoSitePlus, there are still reasons to believe that functionally important methylation events await identification. The high-throughput studies have mainly used trypsin to generate peptides for MS analysis, and there are obviously many cases where a methylation site is contained within a trypsin-generated peptide that, *e.g.*, due to its length or charge state, is not amenable to MS detection. For example, methylation of ATPSc, which was detected in several low-throughput studies, was never reported in any of the high-throughput studies. Also, no dedicated proteomics studies have focused on the identification of methylated proteins in isolated human mitochondria, although such studies have been performed in other organisms, such as budding yeast and trypanosomes (126, 127). Thus, there is a strong need for generating a high-confidence human/mammalian mitochondrial protein methylome by performing protein MS analysis of isolated mitochondria using a panel of different proteases for peptide generation.

### Evolutionary origin of mitochondrial protein MTases

**Figure 3:**
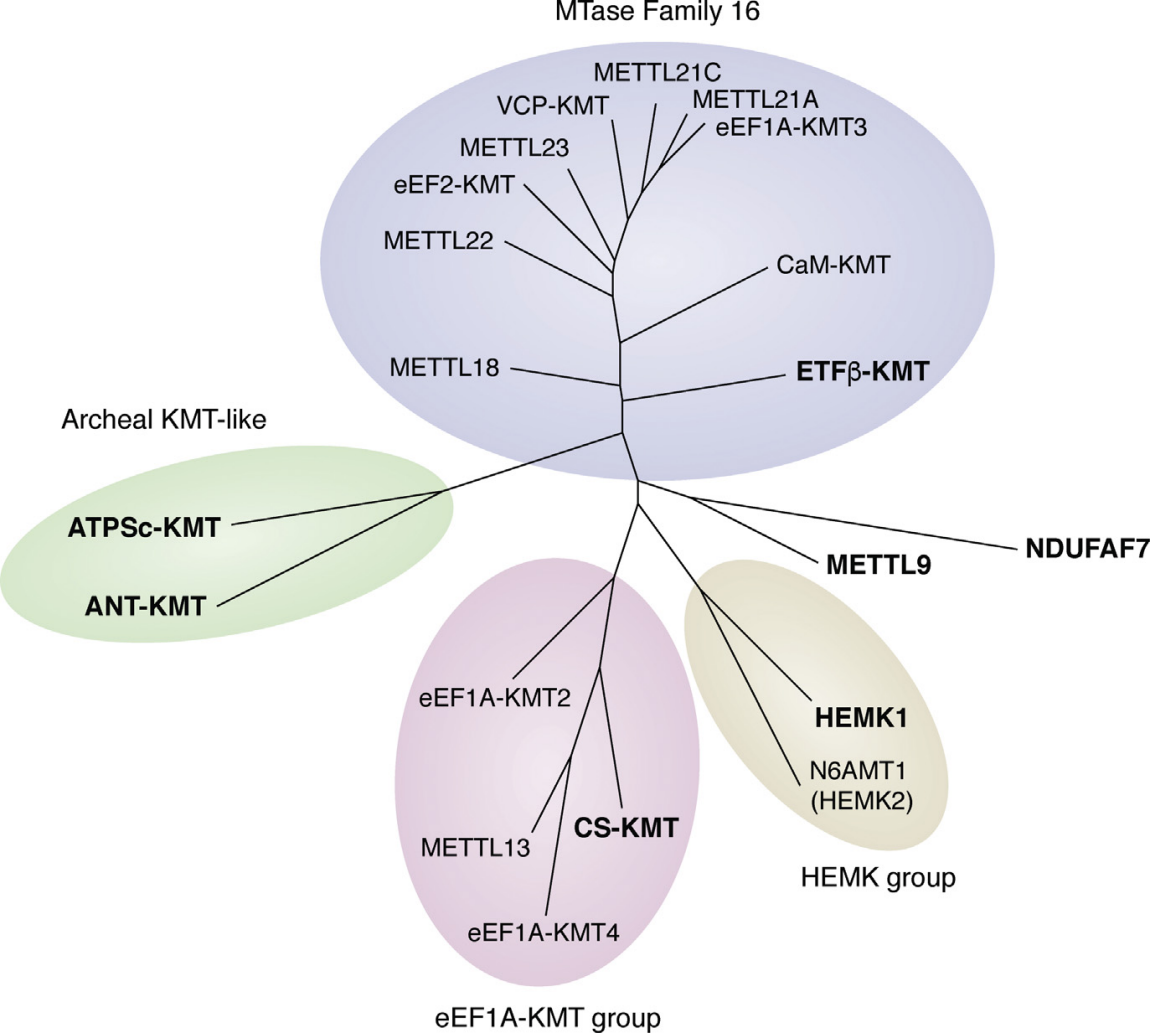
**Phylogenetic grouping of human mitochondrial protein MTases.** An unrooted phylogenetic tree of human mitochondrial protein MTases (bold) and their closest human relatives is shown. *Blue* indicates members of Methyltransferase Family 16, *green* the inter-related ANT-KMT and ATPSc-KMT that are similar to archaeal KMTs, *pink* the group formed by CS-KMT and three eEF1A-specific KMTs, and *beige* HEMK1 and its cytosolic counterpart HEMK2/N6AMT1. First, a sequence alignment of the relevant sequences was generated using MAFFT (133), and then a tree generated and rendered using the PhyML and TREEDYN programs, respectively, both found as part of the Phylogeny.fr package (134, 135, 136). KMT, lysine specific MTase; MTase, methyltransferase.

All the discovered mitochondrial protein MTases in humans belong to the 7BS MTase class, a diverse group of enzymes that collectively methylate a wide range of substrates (17, 18). These enzymes share a similar three-dimensional fold, but, beyond some conserved motifs, the sequence similarity between different MTases is usually low, even between MTases that catalyze the same chemical reaction. For example, several of the 7BS MTases that catalyze lysine methylation show very little sequence similarity to one another, suggesting that KMT activity has arisen independently within the 7BS MTase family at several occasions throughout evolution (19). This is illustrated in Figure 3, which shows an unrooted phylogenetic tree of the human mitochondrial protein MTases and their closest human homologues. ETFβ-KMT belongs to the so-called MTase Family 16, which, in humans, encompasses ten MTases that mainly catalyze lysine methylation (19, 21, 27). Similarly, CS-KMT belongs to a KMT family with four human members, where the three other members all target eukaryotic translation elongation factor 1α (eEF1A) (66), and the RF MTase HEMK1 forms a small group with its cytosolic counterpart HEMK2 (N6AMT1). In contrast, the Arg-specific MTase NDUFAF7 and the His-specific MTase METTL9, as well as the interrelated ATPSc-KMT and ANT-KMT, do not show pronounced similarity to other human MTases. In summary, the mitochondrial protein MTases represent a diverse group of enzymes, some of which are closely related to nonmitochondrial (mainly cytosolic) protein MTases.

According to the endosymbiotic theory, which is now commonly accepted, mitochondria evolved from bacteria taken up by cells, and the bacterial lines that gave rise to mitochondria appear similar to today's α-proteobacteria (128). Interestingly, several of the mitochondrial MTases described here have closely related sequence homologues in α-proteobacteria, and one may speculate that they have a bacterial origin. Moreover, some of the MTases have been shown to modify the same protein on the equivalent residue in bacteria and mitochondria. ETFβ-KMT homologues from the α-proteobacteria *Rhizobium etli* and *Agrobacterium tumefaciens* were shown to methylate the same residues in ETFβ as the human counterpart (64), and Gln methylation of RFs at GGQ motifs by HEMK family enzymes occurs both in bacteria and eukaryotes (129). Also, bioinformatics and structural evidence indicate that NDUFAF7 homologues from α-proteobacteria mediate NDUFS2 methylation (42). The two related MTases ANT-KMT and ATPSc-KMT show similarity to aKMT, a broad-specificity KMT from Archeae, which also has homologues in α-proteobacteria, suggesting a bacterial origin also for these two MTases. In contrast, CS-KMT shows a very limited distribution in eukaryotes; it is found primarily in vertebrates, where it shows a scattered presence, and no bacterial homologues appear to exist. This, taken together with the existence of three CS-KMT paralogues in humans, may suggest that CS-KMT arose from a gene duplication event in a higher eukaryote. Finally, it is difficult to speculate on the evolutionary origin of the His-specific MTase METTL9, since only distant homologues are found in bacteria, and no human paralogues exist.

### Discovering novel mitochondrial protein MTases

So far, seven mitochondrial protein MTases have been described in humans, but given the extent of mitochondrial protein methylation, it is reasonable to assume that more remain to be discovered. Based on the published literature and algorithms for predicting classic MTSs, Rhein *et al*. (39) compiled in 2013 a list of 35 putative and established mitochondrial MTases in humans. In 2017, a similar list was published, but now limited to established and candidate mitochondrial protein MTases, where the latter category comprised uncharacterized MTases with established or putative mitochondrial localization (69). This list, encompassing 11 MTases, included HEMK1, NDUFAF7, METTL9, CS-KMT, and ETFβ-KMT. However, the (then uncharacterized) MTS-less MTases ANT-KMT and ATPSc-KMT were, for obvious reasons, not on the list, underscoring that additional MTS-less mitochondrial MTases may exist. The uncharacterized "orphan" MTases on the list are indeed interesting candidates for mitochondrial protein MTases (69). In particular, the SET-domain MTase SETD9 is the nearest relative of the broad-specificity KMT SETD7 (aka SET7/9) and has also been observed in mitochondria. Also, another SET-domain enzyme, SETD4, which is the closest homologue of the established KMT SETD6, showed, in immunofluorescence experiments, a staining compatible with mitochondrial localization (69, 130). The remaining MTases on the list are not particularly similar to protein MTases, but should still be further investigated, as there are several examples that MTases show activities different from what one would predict from sequence and structural analysis.

In recent years, tremendous progress has been made on identifying novel human protein MTases, but it has also been pointed out that some MTase substrate assignments appear dubious and lack sufficient experimental support (131, 132). However, an important factor in resolving this issue has been the recent availability of MTase KO cells, which now can be readily generated by CRISPR/Cas9 technology. If a protein methylation event is observed in wild-type cells, but not in KO cells lacking a specific MTase, this represents a strong indication that the given MTase is catalyzing the corresponding methylation reaction. Combined with *in vitro* enzymology, this approach allows robust identification of protein MTase substrates; illustratively, many of the recent MTase substrate assignments have been made independently by two or more research groups. Clearly, once a high confidence mitochondrial protein methylome has been established, it will be of great interest to investigate the effect of various MTase KOs on the individual modification events. Since it is highly likely that some of the mitochondrial proteins are methylated prior to import into mitochondria, cytosolic MTases should also be investigated.

## Conclusions and future perspectives

In recent years, considerable progress has been made on the identification of MTases catalyzing mitochondrial protein methylation, particularly in humans. Also, in several cases, it has been demonstrated that methylation of components of the energy production machinery plays important roles in respiration and metabolism. However, several questions remain for future studies. It is necessary to establish a high-quality catalogue of mitochondrial methylation events. Specifically assessing these methylation events in cells/organisms that are deficient in candidate MTases will likely lead to the identification of more MTases involved in mitochondrial methylation. Additionally, it will be interesting to investigate in more detail how methylation affects the molecular properties of the target proteins and to what extent mitochondrial protein methylation actively regulates cellular metabolism.

## Conflict of interest

The authors declare that they have no conflicts of interest with the contents of this article.

## References

[1] Hornbeck P.V., Kornhauser J.M., Tkachev S., Zhang B., Skrzypek E., Murray B., Latham V., Sullivan M. (2012). PhosphoSitePlus: A comprehensive resource for investigating the structure and function of experimentally determined post-translational modifications in man and mouse. Nucleic Acids Res..

[2] Carlson S.M., Gozani O. (2016). Nonhistone lysine methylation in the regulation of cancer pathways. Cold Spring Harb. Perspect. Med..

[3] Rowe E.M., Xing V., Biggar K.K. (2019). Lysine methylation: Implications in neurodegenerative disease. Brain Res..

[4] Bedford M.T. (2007). Arginine methylation at a glance. J. Cell Sci..

[5] Figaro S., Scrima N., Buckingham R.H., Heurgue-Hamard V. (2008). HemK2 protein, encoded on human chromosome 21, methylates translation termination factor eRF1. FEBS Lett..

[6] Kwiatkowski S., Drozak J. (2020). Protein histidine methylation. Curr. Protein Pept. Sci..

[7] Moore K.E., Gozani O. (2014). An unexpected journey: Lysine methylation across the proteome. Biochim. Biophys. Acta.

[8] Greer E.L., Shi Y. (2012). Histone methylation: A dynamic mark in health, disease and inheritance. Nat. Rev. Genet..

[9] Taverna S.D., Li H., Ruthenburg A.J., Allis C.D., Patel D.J. (2007). How chromatin-binding modules interpret histone modifications: Lessons from professional pocket pickers. Nat. Struct. Mol. Biol..

[10] Dimitrova E., Turberfield A.H., Klose R.J. (2015). Histone demethylases in chromatin biology and beyond. EMBO Rep..

[11] Hwang J.W., Cho Y., Bae G.U., Kim S.N., Kim Y.K. (2021). Protein arginine methyltransferases: Promising targets for cancer therapy. Exp. Mol. Med..

[12] Davydova E., Shimazu T., Schuhmacher M.K., Jakobsson M.E., Willemen H.L.D.M., Liu T., Moen A., Ho A.Y.Y., Malecki J., Schroer L., Pinto R., Suzuki T., Gronsberg I.A., Sohtome Y., Akakabe M. (2021). The methyltransferase METTL9 mediates pervasive 1-methylhistidine modification in mammalian proteomes. Nat. Commun..

[13] Kapell S., Jakobsson M.E. (2021). Large-scale identification of protein histidine methylation in human cells. NAR Genom. Bioinform..

[14] Kwiatkowski S., Seliga A.K., Vertommen D., Terreri M., Ishikawa T., Grabowska I., Tiebe M., Teleman A.A., Jagielski A.K., Veiga-da-Cunha M., Drozak J. (2018). SETD3 protein is the actin-specific histidine N-methyltransferase. Elife.

[15] Malecki J.M., Odonohue M.F., Kim Y., Jakobsson M.E., Gessa L., Pinto R., Wu J., Davydova E., Moen A., Olsen J.V., Thiede B., Gleizes P.E., Leidel S.A., Falnes P.O. (2021). Human METTL18 is a histidine-specific methyltransferase that targets RPL3 and affects ribosome biogenesis and function. Nucleic Acids Res..

[16] Wilkinson A.W., Diep J., Dai S., Liu S., Ooi Y.S., Song D., Li T.M., Horton J.R., Zhang X., Liu C., Trivedi D.V., Ruppel K.M., Vilches-Moure J.G., Casey K.M., Mak J. (2019). SETD3 is an actin histidine methyltransferase that prevents primary dystocia. Nature.

[17] Petrossian T.C., Clarke S.G. (2011). Uncovering the human methyltransferasome. Mol. Cell Proteomics.

[18] Schubert H.L., Blumenthal R.M., Cheng X. (2003). Many paths to methyltransfer: A chronicle of convergence. Trends Biochem. Sci..

[19] Falnes P.O., Jakobsson M.E., Davydova E., Ho A.Y., Malecki J. (2016). Protein lysine methylation by seven-β-strand methyltransferases. Biochem. J..

[20] Herz H.M., Garruss A., Shilatifard A. (2013). SET for life: Biochemical activities and biological functions of SET domain-containing proteins. Trends Biochem. Sci..

[21] Cloutier P., Lavallee-Adam M., Faubert D., Blanchette M., Coulombe B. (2013). A newly uncovered group of distantly related lysine methyltransferases preferentially interact with molecular chaperones to regulate their activity. PLoS Genet..

[22] Jakobsson M.E., Moen A., Bousset L., Egge-Jacobsen W., Kernstock S., Melki R., Falnes P.O. (2013). Identification and characterization of a novel human methyltransferase modulating Hsp70 function through lysine methylation. J. Biol. Chem..

[23] Jakobsson M.E., Malecki J.M., Halabelian L., Nilges B.S., Pinto R., Kudithipudi S., Munk S., Davydova E., Zuhairi F.R., Arrowsmith C.H., Jeltsch A., Leidel S.A., Olsen J.V., Falnes P.O. (2018). The dual methyltransferase METTL13 targets N terminus and Lys55 of eEF1A and modulates codon-specific translation rates. Nat. Commun..

[24] Malecki J., Ho A.Y., Moen A., Dahl H.A., Falnes P.O. (2015). Human METTL20 is a mitochondrial lysine methyltransferase that targets the beta subunit of electron transfer flavoprotein (ETFbeta) and modulates its activity. J. Biol. Chem..

[25] Malecki J., Aileni V.K., Ho A.Y., Schwarz J., Moen A., Sorensen V., Nilges B.S., Jakobsson M.E., Leidel S.A., Falnes P.O. (2017). The novel lysine specific methyltransferase METTL21B affects mRNA translation through inducible and dynamic methylation of Lys-165 in human eukaryotic elongation factor 1 alpha (eEF1A). Nucleic Acids Res..

[26] Zoabi M., Zhang L., Li T.M., Elias J.E., Carlson S.M., Gozani O. (2020). Methyltransferase-like 21C (METTL21C) methylates alanine tRNA synthetase at Lys-943 in muscle tissue. J. Biol. Chem..

[27] Kernstock S., Davydova E., Jakobsson M., Moen A., Pettersen S., Maelandsmo G.M., Egge-Jacobsen W., Falnes P.O. (2012). Lysine methylation of VCP by a member of a novel human protein methyltransferase family. Nat. Commun..

[28] Dhayalan A., Kudithipudi S., Rathert P., Jeltsch A. (2011). Specificity analysis-based identification of new methylation targets of the SET7/9 protein lysine methyltransferase. Chem. Biol..

[29] Olsen J.B., Cao X.J., Han B., Chen L.H., Horvath A., Richardson T.I., Campbell R.M., Garcia B.A., Nguyen H. (2016). Quantitative profiling of the activity of protein lysine methyltransferase SMYD2 using SILAC-based proteomics. Mol. Cell Proteomics.

[30] Anderson S., Bankier A.T., Barrell B.G., de Bruijn M.H., Coulson A.R., Drouin J., Eperon I.C., Nierlich D.P., Roe B.A., Sanger F., Schreier P.H., Smith A.J., Staden R., Young I.G. (1981). Sequence and organization of the human mitochondrial genome. Nature.

[31] Pagliarini D.J., Calvo S.E., Chang B., Sheth S.A., Vafai S.B., Ong S.E., Walford G.A., Sugiana C., Boneh A., Chen W.K., Hill D.E., Vidal M., Evans J.G., Thorburn D.R., Carr S.A. (2008). A mitochondrial protein compendium elucidates complex I disease biology. Cell.

[32] Chacinska A., Koehler C.M., Milenkovic D., Lithgow T., Pfanner N. (2009). Importing mitochondrial proteins: Machineries and mechanisms. Cell.

[33] Wiedemann N., Pfanner N. (2017). Mitochondrial machineries for protein import and assembly. Annu. Rev. Biochem..

[34] Omura T. (1998). Mitochondria-targeting sequence, a multi-role sorting sequence recognized at all steps of protein import into mitochondria. J. Biochem..

[35] Zickermann V., Wirth C., Nasiri H., Siegmund K., Schwalbe H., Hunte C., Brandt U. (2015). Structural biology. Mechanistic insight from the crystal structure of mitochondrial complex I. Science.

[36] Elurbe D.M., Huynen M.A. (2016). The origin of the supernumerary subunits and assembly factors of complex I: A treasure trove of pathway evolution. Biochim. Biophys. Acta.

[37] Torija P., Vicente J.J., Rodrigues T.B., Robles A., Cerdan S., Sastre L., Calvo R.M., Escalante R. (2006). Functional genomics in Dictyostelium: MidA, a new conserved protein, is required for mitochondrial function and development. J. Cell Sci..

[38] Carilla-Latorre S., Gallardo M.E., Annesley S.J., Calvo-Garrido J., Grana O., Accari S.L., Smith P.K., Valencia A., Garesse R., Fisher P.R., Escalante R. (2010). MidA is a putative methyltransferase that is required for mitochondrial complex I function. J. Cell Sci..

[39] Rhein V.F., Carroll J., Ding S., Fearnley I.M., Walker J.E. (2013). NDUFAF7 methylates arginine 85 in the NDUFS2 subunit of human complex I. J. Biol. Chem..

[40] Zurita Rendon O., Silva N.L., Sasarman F., Shoubridge E.A. (2014). The arginine methyltransferase NDUFAF7 is essential for complex I assembly and early vertebrate embryogenesis. Hum. Mol. Genet..

[41] Carroll J., Ding S., Fearnley I.M., Walker J.E. (2013). Post-translational modifications near the quinone binding site of mammalian complex I. J. Biol. Chem..

[42] Shahul Hameed U.F., Sanislav O., Lay S.T., Annesley S.J., Jobichen C., Fisher P.R., Swaminathan K., Arold S.T. (2018). Proteobacterial origin of protein arginine methylation and regulation of complex I assembly by MidA. Cell Rep..

[43] Liu F., Wang J., Xing Y., Li T. (2020). Mutation screening of 17 candidate genes in a cohort of 67 probands with early-onset high myopia. Ophthalmic Physiol. Opt..

[44] Wang B., Liu Y., Chen S., Wu Y., Lin S., Duan Y., Zheng K., Zhang L., Gu X., Hong W., Shao H., Zeng X., Sun B., Duan S. (2017). A novel potentially causative variant of NDUFAF7 revealed by mutation screening in a Chinese family with pathologic myopia. Invest. Ophthalmol. Vis. Sci..

[45] Alston C.L., Howard C., Olahova M., Hardy S.A., He L., Murray P.G., O'Sullivan S., Doherty G., Shield J.P., Hargreaves I.P., Monavari A.A., Knerr I., McCarthy P., Morris A.A., Thorburn D.R. (2016). A recurrent mitochondrial p.Trp22Arg NDUFB3 variant causes a distinctive facial appearance, short stature and a mild biochemical and clinical phenotype. J. Med. Genet..

[46] Calvo S.E., Compton A.G., Hershman S.G., Lim S.C., Lieber D.S., Tucker E.J., Laskowski A., Garone C., Liu S., Jaffe D.B., Christodoulou J., Fletcher J.M., Bruno D.L., Goldblatt J., Dimauro S. (2012). Molecular diagnosis of infantile mitochondrial disease with targeted next-generation sequencing. Sci. Transl. Med..

[47] Carroll J., Fearnley I.M., Skehel J.M., Runswick M.J., Shannon R.J., Hirst J., Walker J.E. (2005). The post-translational modifications of the nuclear encoded subunits of complex I from bovine heart mitochondria. Mol. Cell Proteomics.

[48] Petry S., Brodersen D.E., Murphy F.V., Dunham C.M., Selmer M., Tarry M.J., Kelley A.C., Ramakrishnan V. (2005). Crystal structures of the ribosome in complex with release factors RF1 and RF2 bound to a cognate stop codon. Cell.

[49] Nakahigashi K., Kubo N., Narita S., Shimaoka T., Goto S., Oshima T., Mori H., Maeda M., Wada C., Inokuchi H. (2002). HemK, a class of protein methyl transferase with similarity to DNA methyl transferases, methylates polypeptide chain release factors, and hemK knockout induces defects in translational termination. Proc. Natl. Acad. Sci. U. S. A..

[50] Heurgue-Hamard V., Champ S., Engstrom A., Ehrenberg M., Buckingham R.H. (2002). The hemK gene in Escherichia coli encodes the N(5)-glutamine methyltransferase that modifies peptide release factors. EMBO J..

[51] Polevoda B., Span L., Sherman F. (2006). The yeast translation release factors Mrf1p and Sup45p (eRF1) are methylated, respectively, by the methyltransferases Mtq1p and Mtq2p. J. Biol. Chem..

[52] Huh W.K., Falvo J.V., Gerke L.C., Carroll A.S., Howson R.W., Weissman J.S., O'Shea E.K. (2003). Global analysis of protein localization in budding yeast. Nature.

[53] Ishizawa T., Nozaki Y., Ueda T., Takeuchi N. (2008). The human mitochondrial translation release factor HMRF1L is methylated in the GGQ motif by the methyltransferase HMPrmC. Biochem. Biophys. Res. Commun..

[54] Soleimanpour-Lichaei H.R., Kuhl I., Gaisne M., Passos J.F., Wydro M., Rorbach J., Temperley R., Bonnefoy N., Tate W., Lightowlers R., Chrzanowska-Lightowlers Z. (2007). mtRF1a is a human mitochondrial translation release factor decoding the major termination codons UAA and UAG. Mol. Cell.

[55] Young D.J., Edgar C.D., Murphy J., Fredebohm J., Poole E.S., Tate W.P. (2010). Bioinformatic, structural, and functional analyses support release factor-like MTRF1 as a protein able to decode nonstandard stop codons beginning with adenine in vertebrate mitochondria. RNA.

[56] Toogood H.S., Leys D., Scrutton N.S. (2007). Dynamics driving function: New insights from electron transferring flavoproteins and partner complexes. FEBS J..

[57] Frerman F.E. (1988). Acyl-CoA dehydrogenases, electron transfer flavoprotein and electron transfer flavoprotein dehydrogenase. Biochem. Soc. Trans..

[58] Henriques B.J., Katrine Jentoft O.R., Gomes C.M., Bross P. (2021). Electron transfer flavoprotein and its role in mitochondrial energy metabolism in health and disease. Gene.

[59] Davydova E., Ho A.Y., Malecki J., Moen A., Enserink J.M., Jakobsson M.E., Loenarz C., Falnes P.O. (2014). Identification and characterization of a novel evolutionarily conserved lysine-specific methyltransferase targeting eukaryotic translation elongation factor 2 (eEF2). J. Biol. Chem..

[60] Magnani R., Dirk L.M., Trievel R.C., Houtz R.L. (2010). Calmodulin methyltransferase is an evolutionarily conserved enzyme that trimethylates Lys-115 in calmodulin. Nat. Commun..

[61] Rhein V.F., Carroll J., He J., Ding S., Fearnley I.M., Walker J.E. (2014). Human METTL20 methylates lysine residues adjacent to the recognition loop of the electron transfer flavoprotein in mitochondria. J. Biol. Chem..

[62] Toogood H.S., van T.A., Basran J., Sutcliffe M.J., Scrutton N.S., Leys D. (2004). Extensive domain motion and electron transfer in the human electron transferring flavoprotein.medium chain Acyl-CoA dehydrogenase complex. J. Biol. Chem..

[63] Shimazu T., Furuse T., Balan S., Yamada I., Okuno S., Iwanari H., Suzuki T., Hamakubo T., Dohmae N., Yoshikawa T., Wakana S., Shinkai Y. (2018). Role of METTL20 in regulating beta-oxidation and heat production in mice under fasting or ketogenic conditions. Sci. Rep..

[64] Malecki J., Dahl H.A., Moen A., Davydova E., Falnes P.O. (2016). The METTL20 homologue from Agrobacterium tumefaciens is a dual-specificity protein-lysine methyltransferase that targets ribosomal protein L7/L12 and the **β** subunit of electron transfer flavoprotein (ETF**β**). J. Biol. Chem..

[65] Bloxham D.P., Parmelee D.C., Kumar S., Walsh K.A., Titani K. (1982). Complete amino acid sequence of porcine heart citrate synthase. Biochemistry.

[66] Jakobsson M.E., Malecki J., Nilges B.S., Moen A., Leidel S.A., Falnes P.O. (2017). Methylation of human eukaryotic elongation factor alpha (eEF1A) by a member of a novel protein lysine methyltransferase family modulates mRNA translation. Nucleic Acids Res..

[67] Shimazu T., Barjau J., Sohtome Y., Sodeoka M., Shinkai Y. (2014). Selenium-based S-adenosylmethionine analog reveals the mammalian seven-beta-strand methyltransferase METTL10 to be an EF1A1 lysine methyltransferase. PLoS One.

[68] Malecki J., Jakobsson M.E., Ho A.Y.Y., Moen A., Rustan A.C., Falnes P.O. (2017). Uncovering human METTL12 as a mitochondrial methyltransferase that modulates citrate synthase activity through metabolite-sensitive lysine methylation. J. Biol. Chem..

[69] Rhein V.F., Carroll J., Ding S., Fearnley I.M., Walker J.E. (2017). Human METTL12 is a mitochondrial methyltransferase that modifies citrate synthase. FEBS Lett..

[70] Srere P.A. (1974). Controls of citrate synthase activity. Life Sci..

[71] Wiegand G., Remington S.J. (1986). Citrate synthase: Structure, control, and mechanism. Annu. Rev. Biophys. Biophys. Chem..

[72] LaNoue K.F., Bryla J., Williamson J.R. (1972). Feedback interactions in the control of citric acid cycle activity in rat heart mitochondria. J. Biol. Chem..

[73] Smith C.M., Williamson J.R. (1971). Inhibition of citrate synthase by succinyl-CoA and other metabolites. FEBS Lett..

[74] Remington S., Wiegand G., Huber R. (1982). Crystallographic refinement and atomic models of two different forms of citrate synthase at 2.7 and 1.7 A resolution. J. Mol. Biol..

[75] Wiegand G., Remington S., Deisenhofer J., Huber R. (1984). Crystal structure analysis and molecular model of a complex of citrate synthase with oxaloacetate and S-acetonyl-coenzyme A. J. Mol. Biol..

[76] Walker J.E. (2013). The ATP synthase: The understood, the uncertain and the unknown. Biochem. Soc. Trans..

[77] Watt I.N., Montgomery M.G., Runswick M.J., Leslie A.G., Walker J.E. (2010). Bioenergetic cost of making an adenosine triphosphate molecule in animal mitochondria. Proc. Natl. Acad. Sci. U. S. A..

[78] Chen R., Fearnley I.M., Palmer D.N., Walker J.E. (2004). Lysine 43 is trimethylated in subunit C from bovine mitochondrial ATP synthase and in storage bodies associated with batten disease. J. Biol. Chem..

[79] Katz M.L., Christianson J.S., Norbury N.E., Gao C.L., Siakotos A.N., Koppang N. (1994). Lysine methylation of mitochondrial ATP synthase subunit c stored in tissues of dogs with hereditary ceroid lipofuscinosis. J. Biol. Chem..

[80] Walpole T.B., Palmer D.N., Jiang H., Ding S., Fearnley I.M., Walker J.E. (2015). Conservation of complete trimethylation of lysine-43 in the rotor ring of c-subunits of metazoan adenosine triphosphate (ATP) synthases. Mol. Cell Proteomics.

[81] Chu Y., Zhang Z., Wang Q., Luo Y., Huang L. (2012). Identification and characterization of a highly conserved crenarchaeal protein lysine methyltransferase with broad substrate specificity. J. Bacteriol..

[82] Niu Y., Xia Y., Wang S., Li J., Niu C., Li X., Zhao Y., Xiong H., Li Z., Lou H., Cao Q. (2013). A prototypic lysine methyltransferase 4 from archaea with degenerate sequence specificity methylates chromatin proteins Sul7d and Cren7 in different patterns. J. Biol. Chem..

[83] Willemen H.L.D.M., Kavelaars A., Prado J., Maas M., Versteeg S., Nellissen L.J.J., Tromp J., Gonzalez C.R., Zhou W., Jakobsson M.E., Malecki J., Posthuma G., Habib A.M., Heijnen C.J., Falnes P.O. (2018). Identification of FAM173B as a protein methyltransferase promoting chronic pain. PLoS Biol..

[84] Malecki J.M., Willemen H.L.D.M., Pinto R., Ho A.Y.Y., Moen A., Kjonstad I.F., Burgering B.M.T., Zwartkruis F., Eijkelkamp N., Falnes P.O. (2018). Lysine methylation by the mitochondrial methyltransferase FAM173B optimizes the function of mitochondrial ATP synthase. J. Biol. Chem..

[85] Peters M.J., Broer L., Willemen H.L., Eiriksdottir G., Hocking L.J., Holliday K.L., Horan M.A., Meulenbelt I., Neogi T., Popham M., Schmidt C.O., Soni A., Valdes A.M., Amin N., Dennison E.M. (2013). Genome-wide association study meta-analysis of chronic widespread pain: Evidence for involvement of the 5p15.2 region. Ann. Rheum. Dis..

[86] Santiago E., Lopez-Moratalla N., Segovia J.F. (1973). Correlation between losses of mitochondrial ATPase activity and cardiolipin degradation. Biochem. Biophys. Res. Commun..

[87] Pebay-Peyroula E., Dahout-Gonzalez C., Kahn R., Trezeguet V., Lauquin G.J., Brandolin G. (2003). Structure of mitochondrial ADP/ATP carrier in complex with carboxyatractyloside. Nature.

[88] Ruprecht J.J., King M.S., Zogg T., Aleksandrova A.A., Pardon E., Crichton P.G., Steyaert J., Kunji E.R.S. (2019). The molecular mechanism of transport by the mitochondrial ADP/ATP carrier. Cell.

[89] Brenner C., Subramaniam K., Pertuiset C., Pervaiz S. (2011). Adenine nucleotide translocase family: Four isoforms for apoptosis modulation in cancer. Oncogene.

[90] Aquila H., Misra D., Eulitz M., Klingenberg M. (1982). Complete amino acid sequence of the ADP/ATP carrier from beef heart mitochondria. Hoppe Seylers Z Physiol. Chem..

[91] Guo A., Gu H., Zhou J., Mulhern D., Wang Y., Lee K.A., Yang V., Aguiar M., Kornhauser J., Jia X., Ren J., Beausoleil S.A., Silva J.C., Vemulapalli V., Bedford M.T. (2014). Immunoaffinity enrichment and mass spectrometry analysis of protein methylation. Mol. Cell Proteomics.

[92] Malecki J.M., Willemen H.L.D.M., Pinto R., Ho A.Y.Y., Moen A., Eijkelkamp N., Falnes P.O. (2019). Human FAM173A is a mitochondrial lysine-specific methyltransferase that targets adenine nucleotide translocase and affects mitochondrial respiration. J. Biol. Chem..

[93] Beyer K., Klingenberg M. (1985). ADP/ATP carrier protein from beef heart mitochondria has high amounts of tightly bound cardiolipin, as revealed by 31P nuclear magnetic resonance. Biochemistry.

[94] Claypool S.M., Oktay Y., Boontheung P., Loo J.A., Koehler C.M. (2008). Cardiolipin defines the interactome of the major ADP/ATP carrier protein of the mitochondrial inner membrane. J. Cell Biol..

[95] Hedger G., Rouse S.L., Domanski J., Chavent M., Koldso H., Sansom M.S. (2016). Lipid-loving ANTs: Molecular simulations of cardiolipin interactions and the organization of the adenine nucleotide translocase in model mitochondrial membranes. Biochemistry.

[96] Nury H., Dahout-Gonzalez C., Trezeguet V., Lauquin G., Brandolin G., Pebay-Peyroula E. (2005). Structural basis for lipid-mediated interactions between mitochondrial ADP/ATP carrier monomers. FEBS Lett..

[97] DeLange R.J., Glazer A.N., Smith E.L. (1969). Presence and location of an unusual amino acid, epsilon-N-trimethyllysine, in cytochrome c of wheat germ and Neurospora. J. Biol. Chem..

[98] DeLange R.J., Glazer A.N., Smith E.L. (1970). Identification and location of episilon-N-trimethyllysine in yeast cytochromes c. J. Biol. Chem..

[99] DiMaria P., Polastro E., DeLange R.J., Kim S., Paik W.K. (1979). Studies on cytochrome c methylation in yeast. J. Biol. Chem..

[100] Durban E., Nochumson S., Kim S., Paik W.K., Chan S.K. (1978). Cytochrome c-specific protein-lysine methyltransferase from Neurospora crassa. Purification, characterization, and substrate requirements. J. Biol. Chem..

[101] Martzen M.R., McCraith S.M., Spinelli S.L., Torres F.M., Fields S., Grayhack E.J., Phizicky E.M. (1999). A biochemical genomics approach for identifying genes by the activity of their products. Science.

[102] Polevoda B., Martzen M.R., Das B., Phizicky E.M., Sherman F. (2000). Cytochrome c methyltransferase, Ctm1p, of yeast. J. Biol. Chem..

[103] Winter D.L., Abeygunawardena D., Hart-Smith G., Erce M.A., Wilkins M.R. (2015). Lysine methylation modulates the protein-protein interactions of yeast cytochrome C Cyc1p. Proteomics..

[104] Liao H.N., Sherman F. (1979). Yeast cytochrome c-specific protein-lysine methyltransferase: Coordinate regulation with cytochrome c and activities in cyc mutants. J. Bacteriol..

[105] Park K.S., Frost B., Tuck M., Ho L.L., Kim S., Paik W.K. (1987). Enzymatic methylation of *in vitro* synthesized apocytochrome c enhances its transport into mitochondria. J. Biol. Chem..

[106] Brems D.N., Stellwagen E. (1981). The effect of methylation on cytochrome c fragment complementation. J. Biol. Chem..

[107] Kim C.S., Kueppers F., DiMaria P., Farooqui J., Kim S., Paik W.K. (1980). Enzymatic trimethylation of residue-72 lysine in cytochrome c. Effect on the total structure. Biochim. Biophys. Acta.

[108] Pollock W.B., Rosell F.I., Twitchett M.B., Dumont M.E., Mauk A.G. (1998). Bacterial expression of a mitochondrial cytochrome c. Trimethylation of lys72 in yeast iso-1-cytochrome c and the alkaline conformational transition. Biochemistry.

[109] Cherney M.M., Junior C.C., Bowler B.E. (2013). Mutation of trimethyllysine 72 to alanine enhances His79-heme-mediated dynamics of iso-1-cytochrome c. Biochemistry.

[110] Duncan A.L., Robinson A.J., Walker J.E. (2016). Cardiolipin binds selectively but transiently to conserved lysine residues in the rotor of metazoan ATP synthases. Proc. Natl. Acad. Sci. U. S. A..

[111] Fletcher S.C., Coleman M.L. (2020). Human 2-oxoglutarate-dependent oxygenases: Nutrient sensors, stress responders, and disease mediators. Biochem. Soc. Trans..

[112] Haag S., Sloan K.E., Ranjan N., Warda A.S., Kretschmer J., Blessing C., Hübner B., Seikowski J., Dennerlein S., Rehling P., Rodnina M.V., Höbartner C., Bohnsack M.T. (2016). NSUN3 and ABH1 modify the wobble position of mt-tRNAMet to expand codon recognition in mitochondrial translation. EMBO J..

[113] Kawarada L., Suzuki T., Ohira T., Hirata S., Miyauchi K., Suzuki T. (2017). ALKBH1 is an RNA dioxygenase responsible for cytoplasmic and mitochondrial tRNA modifications. Nucleic Acids Res..

[114] Zhang L.S., Xiong Q.P., PeÃ±a P.S., Liu C., Wei J., Le C., Zhang L., Harada B.T., Dai Q., Feng X., Hao Z., Wang Y., Dong X., Hu L., Wang E.D. (2021). ALKBH7-mediated demethylation regulates mitochondrial polycistronic RNA processing. Nat. Cell Biol..

[115] Ougland R., Lando D., Jonson I., Dahl J.A., Moen M.N., Nordstrand L.M., Rognes T., Lee J.T., Klungland A., Kouzarides T., Larsen E. (2012). ALKBH1 is a histone H2A dioxygenase involved in neural differentiation. Stem Cells.

[116] Haws S.A., Leech C.M., Denu J.M. (2020). Metabolism and the epigenome: A dynamic relationship. Trends Biochem. Sci..

[117] Shyh-Chang N., Locasale J.W., Lyssiotis C.A., Zheng Y., Teo R.Y., Ratanasirintrawoot S., Zhang J., Onder T., Unternaehrer J.J., Zhu H., Asara J.M., Daley G.Q., Cantley L.C. (2013). Influence of threonine metabolism on S-adenosylmethionine and histone methylation. Science.

[118] Tibbetts A.S., Appling D.R. (2010). Compartmentalization of Mammalian folate-mediated one-carbon metabolism. Annu. Rev. Nutr..

[119] Larsen S.C., Sylvestersen K.B., Mund A., Lyon D., Mullari M., Madsen M.V., Daniel J.A., Jensen L.J., Nielsen M.L. (2016). Proteome-wide analysis of arginine monomethylation reveals widespread occurrence in human cells. Sci. Signal..

[120] Wang K., Dong M., Mao J., Wang Y., Jin Y., Ye M., Zou H. (2016). Antibody-free approach for the global analysis of protein methylation. Anal. Chem..

[121] Bremang M., Cuomo A., Agresta A.M., Stugiewicz M., Spadotto V., Bonaldi T. (2013). Mass spectrometry-based identification and characterisation of lysine and arginine methylation in the human proteome. Mol. Biosyst..

[122] Uhlmann T., Geoghegan V.L., Thomas B., Ridlova G., Trudgian D.C., Acuto O. (2012). A method for large-scale identification of protein arginine methylation. Mol. Cell Proteomics.

[123] Cao X.J., Arnaudo A.M., Garcia B.A. (2013). Large-scale global identification of protein lysine methylation *in vivo*. Epigenetics.

[124] Wu Z., Cheng Z., Sun M., Wan X., Liu P., He T., Tan M., Zhao Y. (2015). A chemical proteomics approach for global analysis of lysine monomethylome profiling. Mol. Cell Proteomics.

[125] Hart-Smith G., Yagoub D., Tay A.P., Pickford R., Wilkins M.R. (2016). Large scale mass spectrometry-based identifications of enzyme-mediated protein methylation are subject to high false discovery rates. Mol. Cell Proteomics.

[126] Caslavka Zempel K.E., Vashisht A.A., Barshop W.D., Wohlschlegel J.A., Clarke S.G. (2016). Determining the mitochondrial methyl proteome in Saccharomyces cerevisiae using heavy methyl SILAC. J. Proteome Res..

[127] Fisk J.C., Li J., Wang H., Aletta J.M., Qu J., Read L.K. (2013). Proteomic analysis reveals diverse classes of arginine methylproteins in mitochondria of trypanosomes. Mol. Cell Proteomics.

[128] Esser C., Ahmadinejad N., Wiegand C., Rotte C., Sebastiani F., Gelius-Dietrich G., Henze K., Kretschmann E., Richly E., Leister D., Bryant D., Steel M.A., Lockhart P.J., Penny D., Martin W. (2004). A genome phylogeny for mitochondria among alpha-proteobacteria and a predominantly eubacterial ancestry of yeast nuclear genes. Mol. Biol. Evol..

[129] Graille M., Figaro S., Kervestin S., Buckingham R.H., Liger D., Heurgue-Hamard V. (2012). Methylation of class I translation termination factors: Structural and functional aspects. Biochimie.

[130] Faria J.A., Correa N.C., de A.C., de Angelis Campos A.C., Dos Santos Samuel de Almeida R., Rodrigues T.S., de Goes A.M., Gomes D.A., Silva F.P. (2013). SET domain-containing protein 4 (SETD4) is a newly identified cytosolic and nuclear lysine methyltransferase involved in breast cancer cell proliferation. J. Cancer Sci. Ther..

[131] Jakobsson M.E., Moen A., Falnes P.O. (2016). Correspondence: On the enzymology and significance of HSPA1 lysine methylation. Nat. Commun..

[132] Kudithipudi S., Jeltsch A. (2016). Approaches and guidelines for the identification of novel substrates of protein lysine methyltransferases. Cell Chem. Biol..

[133] Katoh K., Misawa K., Kuma K., Miyata T. (2002). Mafft: A novel method for rapid multiple sequence alignment based on fast fourier transform. Nucleic Acids Res..

[134] Chevenet F., Brun C., Banuls A.L., Jacq B., Christen R. (2006). TreeDyn: Towards dynamic graphics and annotations for analyses of trees. BMC Bioinformatics..

[135] Dereeper A., Guignon V., Blanc G., Audic S., Buffet S., Chevenet F., Dufayard J.F., Guindon S., Lefort V., Lescot M., Claverie J.M., Gascuel O. (2008). Phylogeny.fr: Robust phylogenetic analysis for the non-specialist. Nucleic Acids Res..

[136] Guindon S., Dufayard J.F., Lefort V., Anisimova M., Hordijk W., Gascuel O. (2010). New algorithms and methods to estimate maximum-likelihood phylogenies: Assessing the performance of PhyML 3.0. Syst. Biol..

